# Wnt5a promotes ewing sarcoma cell migration through upregulating CXCR4 expression

**DOI:** 10.1186/1471-2407-12-480

**Published:** 2012-10-18

**Authors:** Zhe Jin, Chenghai Zhao, Xiaorui Han, Yaxin Han

**Affiliations:** 1 Department of Orthopedics, The First Hospital of China Medical University, Shenyang, China; 2 Department of Pathophysiology, College of Basic Medical Science, China Medical University, Shenyang, China

## Abstract

**Background:**

As one of the malignant tumors most often affecting children and young adults, Ewing sarcoma (ES) is characterized by early metastasis contributing to unfavorable prognosis. However, the molecular mechanisms responsible for ES metastasis remain poorly understood. In this study, we aimed to explore whether Wnt5a, a putative pro-metastatic factor, plays a role in ES metastasis.

**Methods:**

Expression of Wnt5a and CXCR4 was determined by real-time PCR or Western blot in 15 ES specimens and 4 ES cell lines, A-673, RD-ES, SK-N-MC and SK-ES-1. Expression of Wnt antagonists, SFRP1, SFRP2 and SFRP5, and some components in noncanonical Wnt pathway (p-JNK, p-cJUN and p-PKC) was also analyzed in this study. Methylation status of SFRP1, SFRP2 and SFRP5 was detected by Methylation-specific PCR (MSP). Wnt5a shRNA and pcDNA3.1 SFRP5 vector were used to abrogate Wnt5a expression and overexpress SFRP5 in ES cells, respectively.

**Results:**

Wnt5a expression was positively correlated with CXCR4 expression in ES specimens. Levels of both Wnt5a mRNA and CXCR4 mRNA were significantly higher in specimens from ES patients with metastasis at diagnosis compared with specimens from those without metastasis. Recombinant Wnt5a enhanced CXCR4 expression in ES cells, which was accompanied by increased ES cell migration, whereas Wnt5a shRNA has opposite effects. SFRP5 was methylated and silenced in ES cells, and both recombinant SFRP5 and pcDNA3.1 SFRP5 vector suppressed CXCR4 expression as well as ES cell migration. Wnt5a shRNA and recombinant SFRP5 inhibited phosphorylation of JNK and cJUN, and JNK inhibitor also reduced CXCR4 expression and cell migration in ES cells.

**Conclusions:**

Wnt5a increases ES cell migration via upregulating CXCR4 expression in the absence of Wnt antagonist SFRP5, suggesting that Wnt5a overexpression and SFRP5 deficiency may jointly promote ES metastasis.

## Background

Ewing sarcoma (ES), which mainly affects children and young adults and arises in bone, is characterized by high propensity of metastasis and unfavorable prognosis. So far, there is yet no effective strategy to increase survival rate for ES patients, especially those with metastasis at diagnosis, partially because the molecular mechanisms responsible for ES metastasis remains unclear. As an important representative in noncanonical Wnt family, Wnt5a has been suggested to be a putative pro-metastatic factor by some recent studies [1-4], though, initially, Wnt5a was found to antagonize canonical Wnt/β-catenin pathway, and exert an inhibitory effect on cell proliferation [5,6]. Wnt5a is also expressed in ES [7], however, its role in this tumor has not been explored.

Secreted frizzled-related proteins (SFRPs) are a group of physiological Wnt antagonists, which inhibit Wnt signaling by competing with Wnt receptor Frizzled proteins for Wnt binding. As candidate tumor suppressor genes, SFRPs are frequently methylated and downregulated in human cancers [8-10], which is generally thought to result in excessive activation of Wnt pathways. However, there are few reports documenting the exact Wnt pathways antagonized by SFRPs in human cancers. Neither are there any reports elucidating whether Wnt5a-SFRP5 interaction exists in human cancers, especially in ES, though SFRP5 has been shown to block macrophage activation through inhibition of Wnt5a/JNK signaling in fat tissues [11].

It is well established that chemokine receptor CXCR4 plays a key role in tumor metastasis. Recently, CXCR4 has been shown to be preferentially associated with metastatic ES, suggesting that it may be involved in ES metastasis [12]. In this study, we analyzed the roles of Wnt5a and SFRP5, a putative Wnt5a antagonist, in ES metastasis through investigating CXCR4 expression and ES cell migration. Our study demonstrates for the first time that, via CXCR4 upregulation and JNK activation, Wnt5a-SFRP5 axis may play an important role in ES metastasis.

## Methods

### ES cells and specimens

ES cells, SK-N-MC, SK-ES-1, A-673 and RD-ES, were obtained from American Type Culture Collection (ATCC, Rockville, MD, USA). These cells were cultured in RPMI 1640 supplemented with 10% fetal bovine serum, at 37°C in a humid incubator with 5% CO2. 15 ES specimens were acquired from patients under operation with all their informed consent at the First Hospital of China Medical University, and were frozen in liquid nitrogen immediately after surgical removal. These specimens were divided into two groups: six specimens which were from patients with metastasis at diagnosis were defined as metastatic ESs, and the other 9 specimens were defined as local ESs. This study was performed with the approval of the ethical committee of China Medical University.

### Real-time reverse-transcription PCR

Total RNA was extracted from cells and tissues by Trizol (Takara, Dalian, China) and reverse transcribed by random 9 primer and AMV transcriptase according to the protocol supplied by the manufacturers. Primer sequences for Wnt5a, CXCR4 and GAPDH were described in [1] and [13]. Real-time PCR was carried out using LightCycler DNA Master SYBR Green I Kit in a LightCycler system (LightCycler, Roche Diagnostics). The housekeeping gene glyceraldehyde-3-phosphate dehydrogenase (GAPDH) was used as an internal control. Gene expression was quantified by the comparative CT method, normalizing CT values to GAPDH and calculating relative expression values.

### Western blot

Cell lysates were prepared with sample buffer containing 50mmol/L Tris–HCl (pH 6.8), 100mmol/L DTT, 2% SDS, 0.1% bromophenol blue, and 10% glycerol. 10μg protein of each sample was separated in a 12% sodium dodecyl sulfate (SDS)/ acrylamide gel, and then was transferred to a nylon membrane, which was blocked overnight (4°C in PBS with 0.1% Tween and 10% milk powder). Primary antibodies for Wnt5a, CXCR4, phospho-JNK (p-JNK), phospho-cJun (p-cJun), β-actin and the corresponding secondary antibodies were purchased from Santa Cruz. Phospho-PKC (pan) (βII Ser660) antibody was provided by cell signaling. SFRP5 antibody was provided by Abcam. The human gene β-actin was used as an internal control.

### Methylation-specific PCR and DNA demethylation

DNA was isolated from cells and tissues by a standard phenol/chloroform extraction and ethanol precipitation procedure. Methylation status of SFRP1, SFRP2 and SFRP5 was determined by Genmed MSP Kit (Genmed, Shanghai, China), according to the manufacturer’s protocol. Normal lymphocyte DNA and SssI (NEB, USA)-treated normal lymphocyte DNA served as unmethylated control and methylated control, respectively. Primers for SFRP1, SFRP2 and SFRP5 methylated and unmethylated sequences were described in [14]. A demethylating agent, 5-Aza-2'-deoxycytidine (DAC, 2μmol/L, Sigma) was used to restore SFRP expression in cells with SFRP methylation [9]. In brief, cells were seeded at a density of 3×10^4^ cells/cm^2^ in a 24 well plate on day 0, and exposed to DAC on day 1, 2, and 3. After each treatment, the cells were cultured in fresh medium. Control cells were incubated without the addition of DAC. Cells were harvested on day 4 for experiment.

### RNA interference

Wnt5a shRNA plasmid and nonsilencing control shRNA plasmid were provided by Takala (Dalian, China). Cells were seeded into a 24-well plate at a density of 2×10^5^. On the following day, cells were transfected with shRNA plasmids using Lipofectamine 2000 (Invitrogen, United Kingdom) according to the manufacturer’s instructions. Cells were incubated with shRNA for 48 hours before total RNA was extracted or migration assays were performed.

### Transfection of SFRP5 expression plasmids

The pcDNA3.1 (Invitrogen, Paisley, United Kingdom) SFRP5 vector was made as described in [9]. For transfection experiments, 2×10^5^ cells were plated in a 24-well plate 24 hours before transfection. Lipofectamine 2000 (Invitrogen, Paisley, United Kingdom) was used to perform transfection with 2.0μg pcDNA3.1 SFRP5 vector or 2.0μg pcDNA3.1 empty vector (as control) according to the manufacturer’s protocol.

### Migration assays

Migration of cultured cells was analyzed using transwell chambers (24-well format, 8μm pore). Cells (5×10^5^) were applied to the upper chamber and incubated for 18 hours at 37°C and 5% CO2. Medium supplemented with CXCL12 (100ng/ml, Sigma) was added to the lower chamber as chemoattractant. Migrated cells were stained using 1% toluidine blue after fixation with 100% methanol. For each transwell, the number of migrated cells was counted.

### Statistical analysis

Correlation between Wnt5a expression and CXCR4 expression in ES specimens was analyzed using Spearman’s rank correlation test. Mann–Whitney *U*-test was used to compare mean mRNA levels between metastatic ESs and local ESs. Cell mRNA expression and migration was compared using Student’s *t*-test or one way ANOVA. Statistical analysis was carried out using SPSS version 11.0 (SPSS, Chicago, IL, USA). All *P* values were based on the two-sided statistical analysis, and a *P* value less than 0.05 was considered significant.

## Results

### Differential expression of Wnt5a and CXCR4 in ES tissues and cells

**Figure 1:**
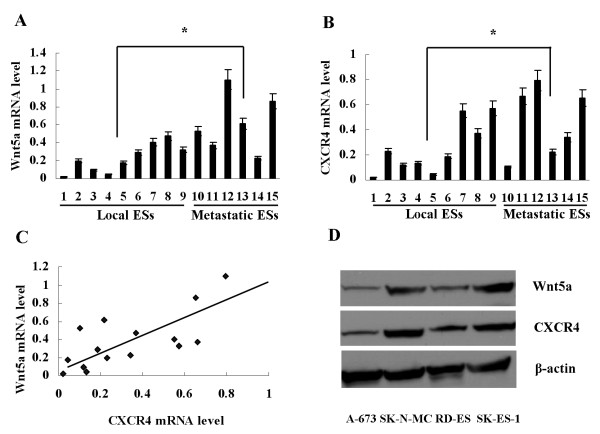
**Differential expression of Wnt5a and CXCR4 in ES tissues and cells. **(**A**) and (**B**) Differential expression of Wnt5a and CXCR4 mRNA was detected in 15 ES specimens by real-time PCR. Both Wnt5a and CXCR4 mean mRNA levels were significantly higher in metastatic ESs compared to local ESs, *P<0.01. (**C**) Wnt5a mRNA level was correlated positively with CXCR4 mRNA level in ES specimens by Spearman’s rank correlation test, *P*<0.01. (**D**) Differential expression of Wnt5a and CXCR4 protein was detected in ES cells by Western blot.

Real-time PCR was used to determine Wnt5a and CXCR4 mRNA expression in 15 ES specimens. Wnt5a mRNA was expressed in all these specimens, however, its level was differential (Figure 1A). Like Wnt5a, CXCR4 mRNA level also varied in these tissues (Figure 1B). However, Wnt5a mRNA level was positively correlated with CXCR4 mRNA level (Figure 1C). In addition, both Wnt5a and CXCR4 mean mRNA levels were significantly higher in metastatic ESs compared with local ESs (Figure 1A, B).

Expression of Wnt5a and CXCR4 was also determined in ES cells. Western blot detection showed a strong expression of Wnt5a and CXCR4 in SK-N-MC and SK-ES-1, whereas a relatively weak expression of these two proteins in A-673 and RD-ES (Figure 1D).

### Upregulation of CXCR4 by Wnt5a in ES cells

**Figure 2:**
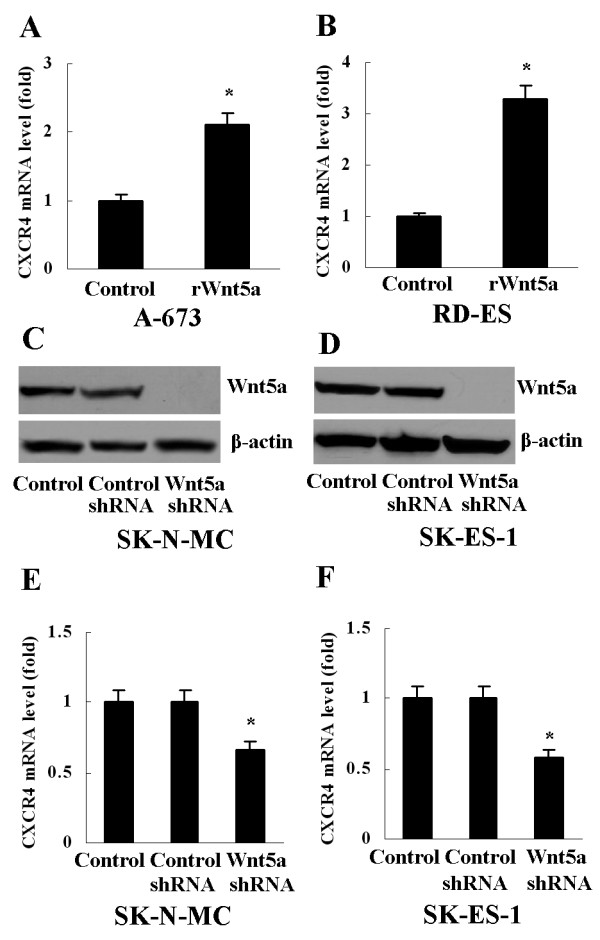
**Upregulation of CXCR4 by Wnt5a in ES cells. **(**A**) and (**B**) Level of CXCR4 mRNA increased in A-673 and RD-ES after treatment with rWnt5a, *P<0.01, *vs* Control (treated with PBS). (**C**) and (**D**) Wnt5a expression was silenced in SK-N-MC and SK-ES-1 cells after transfection with Wnt5a shRNA. (**E**) and (**F**) Level of CXCR4 mRNA decreased in SK-N-MC and SK-ES-1 cells after transfection with Wnt5a shRNA, *P<0.01, *vs* Control and Control shRNA. Data are expressed as mean±SD, n=3.

To explore the correlation of Wnt5a expression with CXCR4 expression in vitro, A-673 and RD-ES, which produce less Wnt5a protein, were treated with recombinant Wnt5a (rWnt5a, 0.1μg/ml, R&D Systems) for 12 hours. Real-time PCR detection showed that level of CXCR4 mRNA increased 2.1 fold in A-673 and 3.3 fold in RD-ES (Figure 2A, B). On the other hand, after transfection with Wnt5a shRNA to silence Wnt5a expression in SK-N-MC and SK-ES-1 (Figure 2C, D), CXCR4 mRNA expression was downregulated significantly, compared with cells with control shRNA or cells without shRNA (Figure 2E, F).

### Promotion of ES cell migration by Wnt5a via CXCR4

**Figure 3:**
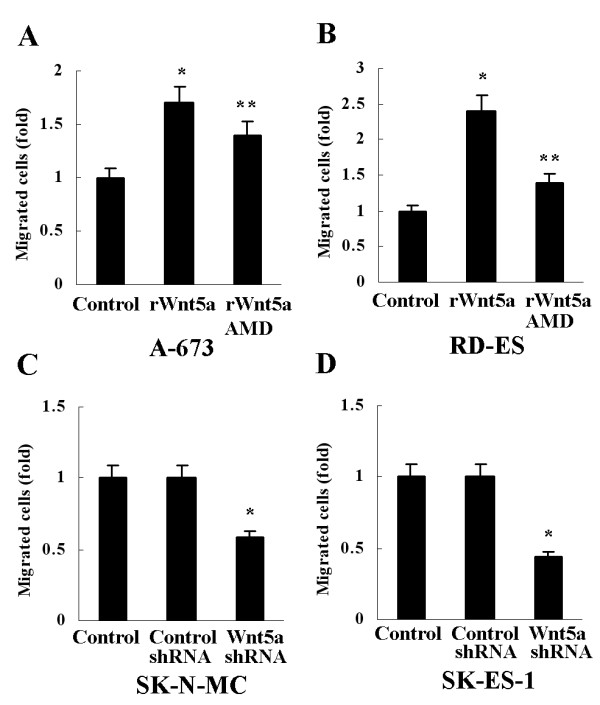
**Promotion of ES cell migration by Wnt5a via CXCR4. **(**A**) and (**B**) The number of migrated A-673 and RD-ES cells increased after treatment with rWnt5a, *P<0.01, *vs* Control. The increase was inhibited when these cells were pre-treated with CXCR4 antagonist AMD 3100, **P<0.01, *vs* rWnt5a. (**C**) and (**D**) The number of migrated SK-N-MC and SK-ES-1 cells decreased after transfection with Wnt5a shRNA, *P<0.01, *vs* Control and Control shRNA. Data are expressed as mean±SD, n=3.

To clarify whether the upregulated CXCR4 expression was functional, migration of ES cells was analyzed in vitro. After treatment with rWnt5a (0.1μg/ml) in A-673 and RD-ES for 12 hours, the number of migrated cells increased 1.7 and 2.4 fold, respectively (Figure 3A, B). However, the induction was almost completely abrogated when these cells were pre-treated with CXCR4 antagonist AMD 3100 (1μg/ml, Sigma) (Figure 3A, B). On the other hand, after Wnt5a shRNA was used to silence Wnt5a expression in SK-N-MC and SK-ES-1, the number of migrated cells decreased significantly, compared with cells with control shRNA or cells without shRNA (Figure 3C, D).

### SFRP5 methylation in ES tissues and cells

**Figure 4:**
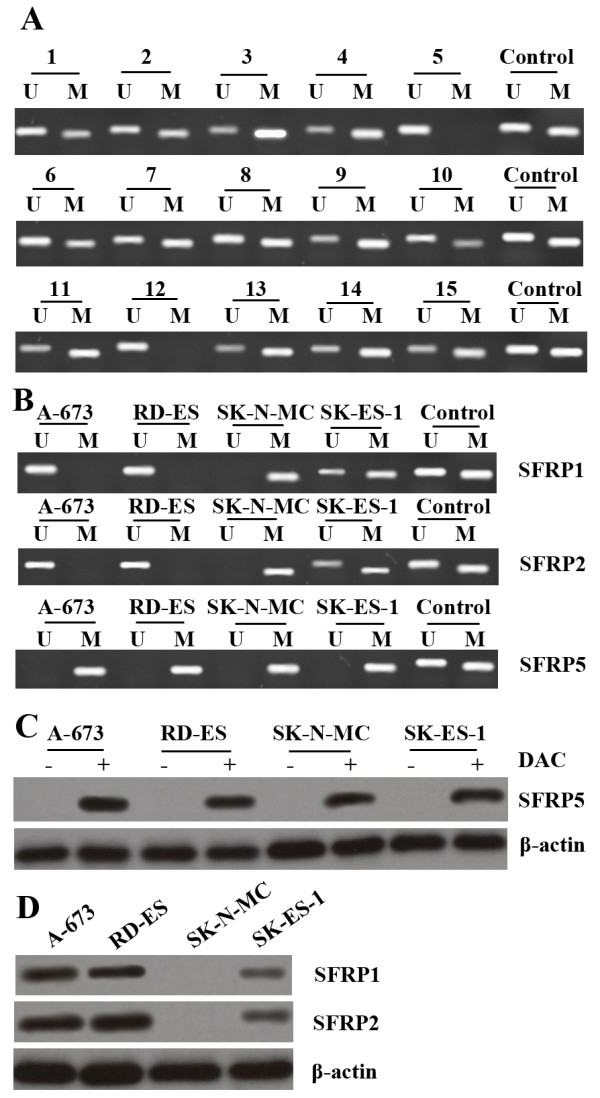
**SFRP5 methylation in Ewing sarcoma tissues and cells. **(**A**) SFRP5 methylation was detected in 13 of 15 ES specimens. (**B**) SFRP1 and SFRP2 were methylated in SK-N-MC and SK-ES-1, while SFRP5 was methylated in all of 4 ES cell lines. (**C**) Expression of SFRP5 protein was restored in 4 ES cells after treatment with DAC. (**D**) SFRP1 and SFRP2 were strongly expressed in A-673 and RD-ES, but weakly expressed in SK-ES-1, and not expressed in SK-N-MC.

Methylation status of SFRP1, SFRP2 and SFRP5 was investigated in 15 ES tissues by MSP. It was found that methylation rate for SFRP1 and SFRP2 was 20% (3/15) and 33% (5/15), respectively, far lower than that for SFRP5 (87%, 13/15,Figure 4A). Methylation status of SFRP1, SFRP2 and SFRP5 was then further determined in 4 ES cell lines, A-673, RD-ES, SK-N-MC and SK-ES-1. As shown by MSP analysis, SFRP5 was methylated in all these cell lines, while SFRP1 and SFRP2 were only methylated in SK-N-MC (completely) and SK-ES-1 (partially) (Figure 4B).

Then Western blot detection showed that SFRP5 was not expressed in all 4 ES cell lines. However, after treatment with DAC, SFRP5 was re-expressed in these cell lines (Figure 4C). SFRP1 and SFRP2 was strongly expressed in A-673 and RD-ES, but weakly expressed in SK-ES-1, and never expressed in SK-N-MC (Figure 4D).

### SFRP5 inhibits CXCR4 expression and ES cell migration

**Figure 5:**
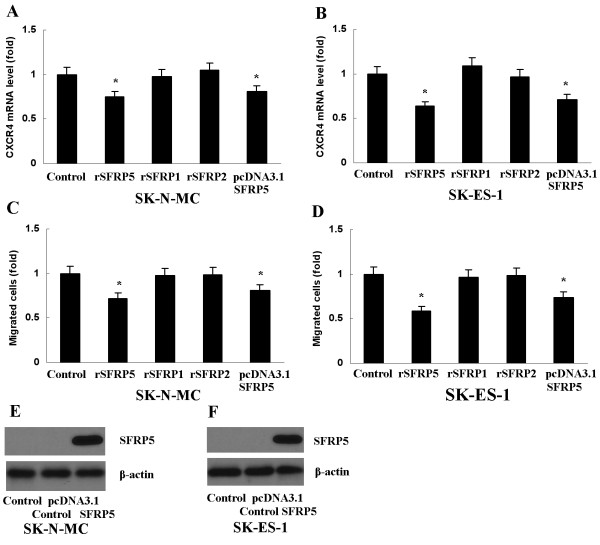
**Inhibition of CXCR4 expression and ES cell migration by SFRP5. **(**A**) and (**B**) Level of CXCR4 mRNA decreased in SK-N-MC and SK-ES-1 cells after treatment with rSFRP5 or transfection with SFRP5 expression vector, *P<0.01, vs Control. (**C**) and (**D**) The number of migrated SK-N-MC and SK-ES-1 cells decreased after treatment with rSFRP5 or transfection with SFRP5 expression vector, *P<0.01, vs Control. (**E**) and (**F**) SFRP5 was expressed in SK-N-MC and SK-ES-1 cells after treatment with pcDNA3.1 SFRP5 vector. Data are expressed as mean±SD, n=3.

To evaluate whether SFRP1, SFRP2 and SFRP5 suppressed CXCR4 expression and ES cell migration, recombinant SFRP1 (rSFRP1), rSFRP2 and rSFRP5 (0.5μg/ml, respectively, R&D Systems) was used to treat SK-ES-1 and SK-N-MC which expressed higher level of Wnt5a, for 12 hours, respectively. It was observed that rSFRP5 reduced CXCR4 expression (Figure 5A, B) and ES cell migration (Figure 5C, D) significantly, whereas rSFRP1 and rSFRP2 did not (Figure 5A-D). To further verify the inhibitory effects of SFRP5, we transfected SK-ES-1 and SK-N-MC with pcDNA3.1 SFRP5 vector (Figure 5E, F). As expected, CXCR4 expression and ES cell migration also decreased (Figure 5A-D).

### Involvement of JNK in Wnt5a-induced ES cell migration

**Figure 6:**
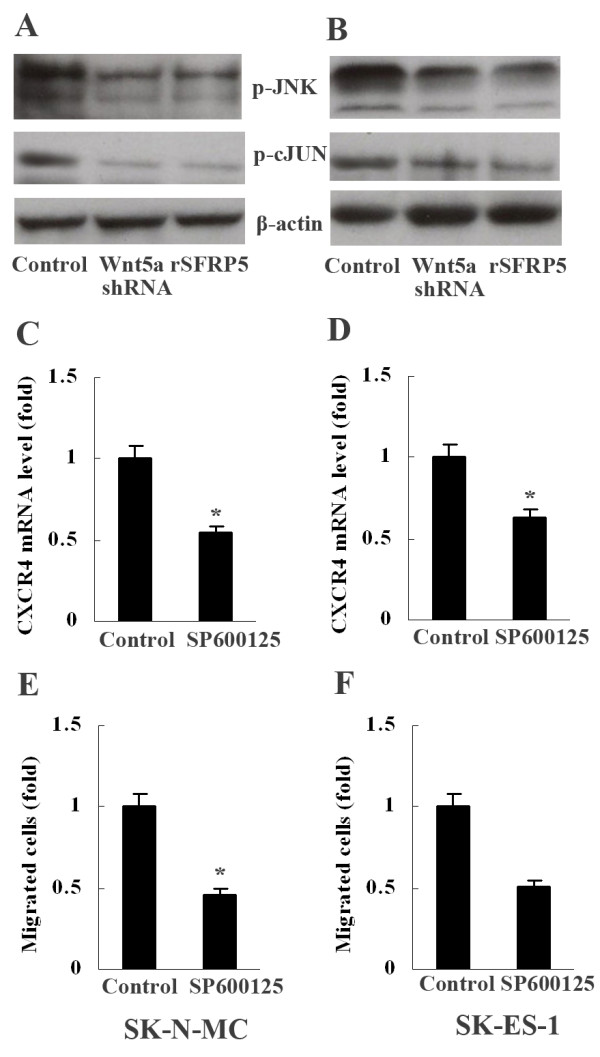
**Involvement of JNK in Wnt5a-induced ES cell migration. **(**A**) and (**B**) Expression of p-JNK and p-cJUN decreased in SK-N-MC and SK-ES-1 cells after treatment with Wnt5a shRNA or rSFRP5. (**C**) and (**D**) Level of CXCR4 mRNA in SK-N-MC and SK-ES-1 cells decreased after treatment with JNK inhibitor SP600125, *P<0.01, *vs* control. (**E**) and (**F**) The number of migrated SK-N-MC and SK-ES-1 cells decreased after treatment with SP600125, *P<0.01, *vs* control. Data are expressed as mean±SD, n=3.

C-Jun N-terminal kinases (JNK) and protein kinase C (PKC) have frequently been observed to be involved in the intracellular signal pathways initiated by extracellular Wnt5a. To explore whether JNK signaling mediated Wnt5a-induced cell migration, phosphorylated JNK (p-JNK) and phosphorylated c-Jun (p-cJun) were detected in ES cells. Western blot detection showed that both Wnt5a shRNA and rSFRP5 (0.5μg/ml) reduced expression of p-JNK and p-cJUN in SK-N-MC and SK-ES-1 (Figure 6A, B). To further determine the role of JNK in Wnt5a-induced ES cell migration, JNK inhibitor SP600125 (10μg/ml, Sigma) was used to treat SK-N-MC and SK-ES-1. It was observed that both CXCR4 expression and ES cell migration were suppressed remarkably (Figure 6C-F).

**Figure 7:**
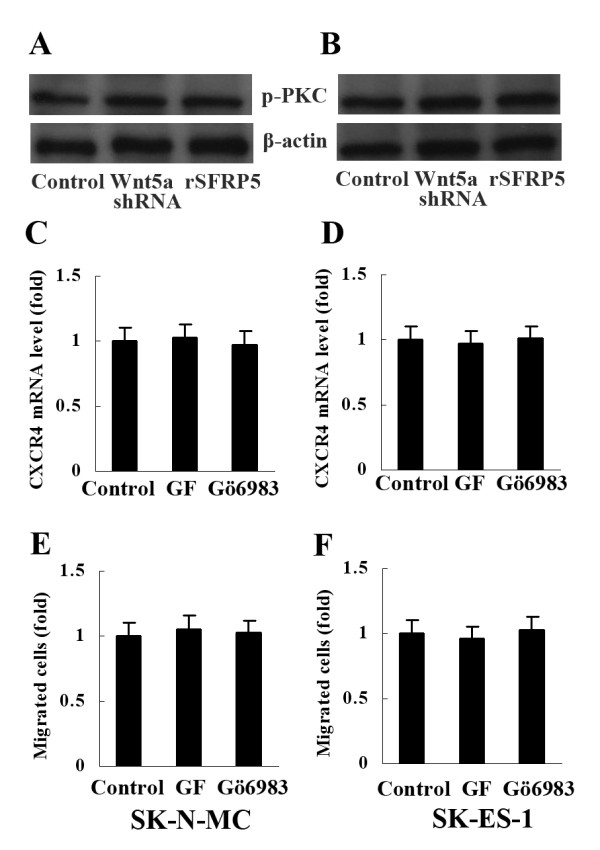
**PKC was not involved in Wnt5a-induced ES cell migration. **(**A**) and (**B**) Neither Wnt5a shRNA nor rSFRP5 reduced the expression of phosphorylated PKC in SK-N-MC and SK-ES-1. (**C**-**F**) PKC inhibitors GF109203X and Gö6983 had no effect on CXCR4 expression and cell migration. GF: GF109203X. Data are expressed as mean±SD, n=3.

We also determined whether Wnt/PKC pathway was involved in ES cell migration. As shown by Western blot analysis, neither Wnt5a shRNA nor rSFRP5 reduced the expression of phosphorylated PKC (p-PKC) in SK-N-MC and SK-ES-1 (Figure 7A, B). In addition, PKC inhibitors bisindolylmaleimide I (GF109203X, 10μg/ml, Santa Cruz) and Gö6983 (10μg/ml, Santa Cruz) had no effect on CXCR4 expression and ES cell migration (Figure 7C-F).

## Discussion

Despite the application of modern multimodal therapeutic regimens, ES is still characterized with a high mortality, which could be mainly attributed to the development of distant metastasis [15]. However, up till now, mechanisms underlying ES metastasis still remain largely unknown, and few factors involved in this process have been identified [16-18]. Our investigation of the role of Wnt5a in ES metastasis points to the following findings: First, ES specimens from patients with metastasis at diagnosis expressed more Wnt5a than those from patients without metastasis. Second, treatment with recombinant Wnt5a significantly increased ES cell migration. Third, Wnt5a knockdown by Wnt5a shRNA notably reduced ES cell migration. Finally, treatment with either rSFRP5 or SFRP5 expression vector also remarkably suppressed ES cell migration. Taken together, our report provides all lines of evidence that Wnt5a may be a pro-metastatic factor in ES.

This study identifies CXCR4 as a downstream component in Wnt5a pathway in ES. In fact, correlation of Wnt5a with CXCR4 was firstly observed in ES specimens. Then, it was found that recombinant Wnt5a enhanced CXCR4 expression in ES cells, whereas Wnt5a shRNA exerted a negative effect on CXCR4 expression. Furthermore, SFRP5 was also shown to inhibit CXCR4 expression in ES. More importantly, CXCR4 antagonist AMD 3100 was found to remarkably inhibit Wnt5a-induced ES cell migration. Wnt5a-induced CXCR4 upregulation may be responsible for, at least in part, the lung metastasis in ES, because the lung is one of the richest sources of chemokine CXCL12, the ligand for CXCR4. Our finding is consistent with another report that Wnt5a was required for CXCL12-mediated T-cell migration and the sustained expression of CXCR4 in T cells [19].

Our study is the first report to provide evidence that SFRP5 methylation may be a common phenomenon in ES, supported by the findings that 87 percent of ES specimens and all four ES cell lines tested in this study harbor SFRP5 methylation. Furthermore, we detected the absence of SFRP5 expression in all four ES cell lines, and showed its presence after these cell lines were treated with demethylating agent DAC, indicating SFRP5 methylation is responsible for SFRP5 downregulation. Our study also raises the possibility that SFRP5 expression deficiency may facilitate Wnt5a signaling in ES, based on the findings that both rSFRP5 and SFRP5 expression vector blocked Wnt5a-induced CXCR4 expression and cell migration. The present report eliminates the possibility that SFRP1 and SFRP2 are involved in Wnt5a signaling in ES, supported by the evidence that both SFRP1 and SFRP2, unlike SFRP5, are infrequently methylated in ES, and neither of them has an inhibitory effect on Wnt5a-induced CXCR4 expression and cell migration in SK-N-MC and SK-ES-1, though they both are also methylated and underexpressed in these two cell lines.

Studies have shown that both JNK and PKC can mediate Wnt5a signaling in some pathological processes, including inflammation and carcinogenesis [1,4,11]. In the present study, expression of p-JNK and p-cJUN was suppressed significantly when ES cells were treated with either Wnt5a shRNA to abrogate Wnt5a expression or rSFRP5 to block Wnt5a action. Furthermore, treatment with JNK inhibitor SP600125 remarkably inhibited CXCR4 expression as well as ES cell migration. These results collectively indicate that JNK mediates Wnt5a-induced ES cell migration, which is consistent with another report that JNK mediated Wnt5a-dependent prostate cancer cell migration [4]. On the contrary, our study has not demonstrated the involvement of Wnt5a/PKC pathway in ES metastasis, though it is well established that this pathway plays a crucial role in melanoma invasion [1,20]. Interestingly, it has been shown that both JNK and PKC are involved in Wnt5a-induced gastric cancer cell invasion and migration through induction of Laminin gamma 2 [21]. The above findings clearly indicate that the intracellular signals mediating extracellular Wnt5a are tissue-specific.

In summary, our study demonstrates that Wnt5a enhances CXCR4 expression via activation of JNK in SFRP5-negative ES cells, which is accompanied by increased ES cell migration. Another result from our study is that both rSFRP5 and SFRP5 expression vector effectively blocked Wnt5a-induced ES cell migration. These findings clearly points to a positive role of Wnt5a in ES metastasis, as well as a defensive role of SFRP5 in ES progression. In addition, based on the findings that both JNK inhibitor and CXCR4 antagonist had significant oppressive effects on Wnt5a-induced ES cell migration, we speculate that JNK and CXCR4 may be compelling candidates to be additional potential therapeutic targets for Wnt5a-dependent ES metastasis.

## Conclusions

Wnt5a increases ES cell migration via upregulating CXCR4 expression in the absence of Wnt antagonist SFRP5, suggesting that Wnt5a overexpression and SFRP5 deficiency may jointly promote ES metastasis.

## Competing interests

The authors declare that they have no competing interests.

## Authors’ contributions

JZ designed the study, analyzed and interpreted the data, and drafted the manuscript. ZC carried out Western analysis and cell transfection. HX performed PCR analysis. HY was engaged in cell migration assay and statistical analysis. All authors read and approved the final manuscript.

## Pre-publication history

The pre-publication history for this paper can be accessed here:

http://www.biomedcentral.com/1471-2407/12/480/prepub
